# An open dataset of *Plasmodium vivax* genome variation in 1,895 worldwide samples

**DOI:** 10.12688/wellcomeopenres.17795.1

**Published:** 2022-04-14

**Authors:** MalariaGEN, Ishag Adam, Mohammad Shafiul Alam, Sisay Alemu, Chanaki Amaratunga, Roberto Amato, Voahangy Andrianaranjaka, Nicholas M Anstey, Abraham Aseffa, Elizabeth Ashley, Ashenafi Assefa, Sarah Auburn, Bridget E Barber, Alyssa Barry, Dhelio Batista Pereira, Jun Cao, Nguyen Hoang Chau, Kesinee Chotivanich, Cindy Chu, Arjen M. Dondorp, Eleanor Drury, Diego F. Echeverry, Berhanu Erko, Fe Espino, Rick Fairhurst, Abdul Faiz, María Fernanda Villegas, Qi Gao, Lemu Golassa, Sonia Goncalves, Matthew J Grigg, Yaghoob Hamedi, Tran Tinh Hien, Ye Htut, Kimberly J Johnson, Nadira Karunaweera, Wasif Khan, Srivicha Krudsood, Dominic P Kwiatkowski, Marcus Lacerda, Benedikt Ley, Pharath Lim, Yaobao Liu, Alejandro Llanos-Cuentas, Chanthap Lon, Tatiana Lopera-Mesa, Jutta Marfurt, Pascal Michon, Olivo Miotto, Rezika Mohammed, Ivo Mueller, Chayadol Namaik-larp, Paul N Newton, Thuy-Nhien Nguyen, Francois Nosten, Rintis Noviyanti, Zuleima Pava, Richard D Pearson, Beyene Petros, Aung P Phyo, Ric N Price, Sasithon Pukrittayakamee, Awab Ghulam Rahim, Milijaona Randrianarivelojosia, Julian C Rayner, Angela Rumaseb, Sasha V Siegel, Victoria J Simpson, Kamala Thriemer, Alberto Tobon-Castano, Hidayat Trimarsanto, Marcelo Urbano Ferreira, Ivan D Vélez, Sonam Wangchuk, Thomas E Wellems, Nicholas J White, Timothy William, Maria F Yasnot, Daniel Yilma

**Affiliations:** 1 Faculty of Medicine, University of Khartoum, Khartoum, Sudan; 2 Infectious Diseases Division, International Centre for Diarrheal Diseases Research, Bangladesh (ICDDR,B), Dhaka, Bangladesh; 3 Armauer Hansen Research Unit (AHRI), Addis Ababa, Ethiopia; 4 Addis Ababa University, Addis Ababa, Ethiopia; 5 MilliporeSigma (Bioreliance), Rockville, USA; 6 National Institute of Allergy and Infectious Diseases (NIAID), NIH, Bethesda, USA; 7 Wellcome Sanger Institute, Hinxton, UK; 8 Université d'Antananarivo, Antananarivo, Madagascar; 9 Global and Tropical Health Division, Menzies School of Health Research and Charles Darwin University, Darwin, Australia; 10 Lao-Oxford-Mahosot Hospital-Wellcome Trust Research Unit, Microbiology Laboratory, Mahosot Hospital, Vientiane, Lao People's Democratic Republic; 11 Centre for Tropical Medicine and Global Health, Nuffield Department of Medicine, University of Oxford, Oxford, UK; 12 Ethiopian Public Health Institute, Addis Ababa, Ethiopia; 13 Mahidol‐Oxford Tropical Medicine Research Unit, Mahidol University, Bangkok, Thailand; 14 Menzies School of Health Research, Darwin, Australia; 15 QIMR Berghofer Medical Research Institute, Brisbane, Australia; 16 Walter and Eliza Hall Institute, Parkville, Australia; 17 Deakin University, Geelong, Australia; 18 Burnet Institute, Melbourne, Australia; 19 Centro de Pesquisa em Medicina Tropical de Rondônia, Porto Velho, Brazil; 20 National Health Commission Key Laboratory of Parasitic Disease Control and Prevention, Jiangsu Provincial Key Laboratory on Parasite and Vector Control Technology, Jiangsu Institute of Parasitic Diseases, Wuxi, China; 21 Center for Global Health, School of Public Health, Nanjing Medical University, Nanjing, China; 22 Oxford University Clinical Research Unit, Hospital for Tropical Diseases, Ho Chi Minh City, Vietnam; 23 Mahidol University, Bangkok, Thailand; 24 Shoklo Malaria Research Unit, Mahidol-Oxford Tropical Medicine Research Unit, Faculty of Tropical Medicine, Mahidol University, Mae Sot, Thailand; 25 Departamento de Microbiologia, Facultad de Salud, Universidad del Valle, Cali, Colombia; 26 Aklilu Lemma Institute of Pathobiology, Addis Ababa University, Addis Ababa, Ethiopia; 27 Research Institute for Tropical Medicine, Department of Health, Manila, Philippines; 28 National Institutes of Health (NIH), Bethesda, USA; 29 Dev Care Foundation, Dhaka, Bangladesh; 30 Centro de Investigaciones Clinicas, Cali, Colombia; 31 Infectious and Tropical Diseases Research Center, Hormozgan University of Medical Sciences, Bandar Abbas, Iran; 32 Department of Medical Research, Yangon, Myanmar; 33 University of Colombo, Colombo, Sri Lanka; 34 School of Public Health, Harvard University, Boston, USA; 35 Instituto de Pesquisa Clínica Carlos Borborema, Fundação de Medicina Tropical Dr Heitor Vieira Dourado, Manaus, Brazil; 36 Instituto Leônidas & Maria Deane, Fundação Oswaldo Cruz, Manaus, Brazil; 37 Parsons Corporation, Walter Reed Army Institute of Research (WRAIR), Silver Spring, USA; 38 Cayetano Heredia University, Lima, Peru; 39 National Institute of Allergy and Infectious Diseases, Phnom Penh, Cambodia; 40 Universidad de Antioquia, Medellin, Colombia; 41 National University of Vanuatu, Port-Vila, Vanuatu; 42 Department of Internal Medicine, University of Gondar, Gondar, Ethiopia; 43 Umphang Hospital, Tak, Thailand; 44 Eijkman Institute for Molecular Biology, Jakarta, Indonesia; 45 Centro Internacionale de Entrenamiento e Investigaciones Medicas, Cali, Colombia; 46 Shoklo Malaria Research Unit, Bangkok, Thailand; 47 Nangarhar Medical Faculty, Nangarhar University, Ministry of Higher Education, Jalalabad, Afghanistan; 48 Institut Pasteur de Madagascar, Antananarivo, Madagascar; 49 Universités d'Antananarivo et de Mahajanga, Antananarivo, Madagascar; 50 Cambridge Institute for Medical Research, University of Cambridge, Cambridge, UK; 51 Universidade de São Paulo, São Paulo, Brazil; 52 Institute of Hygiene and Tropical Medicine, NOVA University of Lisbon, Lisbon, Portugal; 53 University of Antioquia, Medellin, Colombia; 54 Royal Center for Disease Control, Department of Public Health, Ministry of Health, Thimphu, Bhutan; 55 Clinical Research Centre, Queen Elizabeth Hospital, Sabah, Malaysia; 56 Infectious Diseases Society Sabah-Menzies School of Health Research Clinical Research Unit, Kota Kinabalu, Sabah, Malaysia; 57 Grupo de Investigaciones Microbiológicas y Biomédicas de Córdoba-GIMBIC, Universidad de Córdoba, Monteria, Colombia; 58 Jimma University, Jimma, Ethiopia

**Keywords:** malaria, plasmodium vivax, genomics, data resource, genomic epidemiology

## Abstract

This report describes the MalariaGEN Pv4 dataset, a new release of curated genome variation data on 1,895 samples of *Plasmodium vivax* collected at 88 worldwide locations between 2001 and 2017. It includes 1,370 new samples contributed by MalariaGEN and VivaxGEN partner studies in addition to previously published samples from these and other sources. We provide genotype calls at over 4.5 million variable positions including over 3 million single nucleotide polymorphisms (SNPs), as well as short indels and tandem duplications. This enlarged dataset highlights major compartments of parasite population structure, with clear differentiation between Africa, Latin America, Oceania, Western Asia and different parts of Southeast Asia. Each sample has been classified for drug resistance to sulfadoxine, pyrimethamine and mefloquine based on known markers at the *dhfr*, *dhps* and *mdr1* loci. The prevalence of all of these resistance markers was much higher in Southeast Asia and Oceania than elsewhere. This open resource of analysis-ready genome variation data from the MalariaGEN and VivaxGEN networks is driven by our collective goal to advance research into the complex biology of *P. vivax* and to accelerate genomic surveillance for malaria control and elimination.

## Background

*Plasmodium vivax* is the second most common cause of human malaria, with an extensive geographical range ^1, 2^ *. P. vivax* has a number of biological features that distinguish it from the more widely studied *P. falciparum*. Importantly, *P. vivax* establishes dormant forms in the liver that are refractory to most antimalarial drugs, resulting in relapsing infections that represent a major challenge to malaria elimination ^3– 5^. Additionally, a cryptic endosplenic life-cycle results in a large hidden splenic reservoir of *P. vivax* parasites ^6, 7^ which sustains a high prevalence of low-density asymptomatic blood stage infections. *P. vivax* is uncommon in much of sub-Saharan Africa, and this is thought to be primarily due to the high frequency of the Duffy negative blood group that inhibits invasion by this species, although the parasite can sometimes break through this protection by unknown mechanisms ^8^. Clinical disease occurs at lower circulating parasite densities for *P. vivax* than for *P. falciparum*, making the detection and characterisation of infections considerably more difficult ^3^. Analysis of *P. vivax* genome variation is technically challenging for a number of reasons, particularly the difficulty of getting high quality sequence data due to low parasite density in clinical blood samples. An additional challenge is high levels of within-host diversity in some peripheral blood samples, that can be due to either superinfection or cotransmission ^9^ and is exacerbated by relapsing infections or spillover from extravascular reservoirs. It is widely accepted that *P. vivax* is likely to be more challenging to eliminate than *P. falciparum*, and indeed, in countries approaching elimination the proportion of malaria due to *vivax* has increased ^1, 2^.

Here we report a new data release from the MalariaGEN *Plasmodium vivax* Genome Variation Project which was established in 2010 to enable malaria researchers to integrate parasite genome sequencing into clinical and epidemiological studies of *P. vivax* ( https://www.malariagen.net/parasite/p-vivax-genome-variation). Genome sequencing was performed at the Wellcome Sanger Institute and a standardised analysis pipeline was used for variant discovery and genotyping. Sequence data and genotype calls were returned to partners for use in their own analyses and publications in line with MalariaGEN’s guiding principles on equitable data sharing ^10^. Each data release to partners is given a version number and the current version is called Pv4.

The Pv4 dataset comprises 1,895 samples from 27 countries, most of which were sequenced at the Wellcome Sanger Institute. Of the 1,306 samples that have not previously been published, the majority came from a collaboration between MalariaGEN and the VivaxGEN network ( http://menzies.edu.au/vivaxGEN) led by Menzies School of Health Research, and from a multicentre clinical trial led by GlaxoSmithKline ^11, 12^. We have also included 292 samples from a previous MalariaGEN publication ^13^ and 297 samples from previously published studies by other research groups ^14– 16^. All samples have been reanalysed using a standardised pipeline to minimise potential artefacts arising from different sequencing protocols.

To make these data as useful as possible to other researchers, we provide curated genotype calls on millions of SNPs, indels, and tandem duplications. We have classified samples for evidence of resistance to sulfadoxine, pyrimethamine and mefloquine based on known genetic markers. Each sample is evaluated for within-host diversity and for its location in the global parasite population structure. This new data release increases the sample size of *P. vivax* genome variation data by more than threefold, and it provides an open resource of curated, analysis-ready data with many potential applications both for basic scientific research and in building genomic surveillance tools for malaria control and elimination.

## Resource data

The 1,895 samples in the Pv4 dataset were collected from 88 locations in 27 countries in Asia, Oceania, Latin America and Africa, mostly between 2001 and 2017 ( Table 1– Table 4). 1,026 samples were collected by the VivaxGEN network, a global collaboration using translational genomics to develop new molecular surveillance tools to support the elimination of *P. vivax*. A further 357 samples were collected as part of drug safety and efficacy trials led by GlaxoSmithKline in Latin America, Asia and Africa ^11, 12^. There were 215 samples from other MalariaGEN partner studies, the details of which can be found in Table 3. Finally, we have integrated 297 previously-published samples that were sequenced by the Broad Institute, the University of North Carolina at Chapel Hill and the Wellcome Sanger Institute as part of other research collaborations ^14– 16^. Since the dataset included samples from multiple sequencing labs with different protocols it was necessary to perform systematic curation to minimise the introduction of biases.

**Table 1.** Count of samples in the dataset. Countries are grouped into seven geographic regions based on their geographic and genetic characteristics. For each country, the table reports: the number of distinct sampling locations; the total number of samples sequenced; the number of high-quality samples; the number of high-quality samples included in the analysis; and the percentage of samples collected between 2015–2017, the most recent sampling period in the dataset. 70 samples are from countries that are genetically distinct from those from the seven regions, and a further 48 samples from Bangkok could not be assigned to either the WSEA or ESEA region. These 118 samples (of which 41 passed QC) are classified as unassigned. The breakdown by site is reported in Table 2 and the list of contributing studies in Table 3 and Table 4.

Region	Country	Sampling locations	Sequenced samples	QC pass samples	Analysis set samples	% analysis samples 2015–2017
***Latin America (LAM)***	**Brazil**	6	71	21	21	24%
	**Colombia**	12	112	67	67	39%
	**El Salvador**	1	2	1	1	0%
	**Mexico**	5	20	20	20	0%
	**Nicaragua**	1	1	1	1	0%
	**Panama**	1	1	1	1	0%
	**Peru**	6	123	48	48	15%
***Africa (AF)***	**Ethiopia**	7	203	137	137	39%
***Western Asia (WAS)***	**Afghanistan**	2	250	36	36	81%
	**India**	4	14	5	5	0%
	**Iran**	1	15	5	5	0%
	**Sri Lanka**	1	2	1	1	0%
***Western Southeast*** ***Asia (WSEA)***	**Western Thailand**	5	141	127	127	7%
***Eastern Southeast*** ***Asia (ESEA)***	**Cambodia**	7	236	172	172	28%
	**Northeastern Thailand**	2	3	2	2	0%
	**Vietnam**	6	139	103	103	88%
***Maritime Southeast*** ***Asia (MSEA)***	**Malaysia**	2	109	73	73	0%
	**Philippines**	1	6	3	3	100%
***Oceania (OCE)***	**Indonesia**	2	282	191	191	18%
	**Papua New Guinea**	4	47	17	17	0%
***Unassigned samples*** ***(unassigned)***	**Bangladesh**	1	28	6	0	
	**Bhutan**	1	9	2	0	
	**China**	1	5	5	0	
	**Madagascar**	3	4	4	0	
	**Mauritania**	1	1	1	0	
	**Myanmar**	2	9	8	0	
	**North Korea**	1	1	1	0	
	**Sudan**	1	13	4	0	
	**Thailand (Bangkok)**	1	48	10	0	
***Total***		88	1,895	1,072	1,031	30%

**Table 2.** Breakdown of analysis set samples by geography. Sites are divided into seven regions as described in the main text. Note that samples from Pakchong and Sisaket in eastern Thailand have been assigned to the Eastern SE Asia (ESEA) region whereas samples from other regions in Thailand have been assigned to the Western SE Asia (WSEA) region. 41 samples that passed QC but were not assigned to one of the seven regions have been excluded from analyses.

Region	Country	First-level administrative division	Site	Sequenced samples	Analysis set samples
***LAM***	**Brazil**	**Brazil**	**Brazil**	6	4
		**Brazil: Acre**	**Acrelândia**	7	1
			**Plácido de Castro**	13	1
		**Brazil: Amazonas**	**Manaus**	37	14
		**Brazil: Para**	**Belem**	1	1
		**Brazil: Rondonia**	**Porto Velho**	7	0
	**Colombia**	**Colombia**	**Colombia**	3	2
		**Colombia: Antioquia**	**Antioquia**	8	2
		**Colombia: Bolivar**	**Bolivar**	1	0
		**Colombia: Choco**	**Choco**	26	13
			**Pichimá**	1	0
		**Colombia: Cordoba**	**Cordoba**	3	3
			**Córdoba**	1	1
			**Tierralta**	43	37
		**Colombia: Narino**	**Tumaco**	2	2
		**Colombia: Risaralda**	**Santa Cecilia**	16	3
		**Colombia: Valle del Cauca**	**Buenaventura**	3	3
			**Cali**	5	1
	**El Salvador**	**El Salvador**	**El Salvador**	2	1
	**Mexico**	**Mexico: Chiapas**	**Carrillo**	1	1
			**Frontera Hidalgo**	1	1
			**Huehuetán**	1	1
			**Tapachula**	16	16
			**Tuxtla Chico**	1	1
	**Nicaragua**	**Nicaragua**	**Nicaragua**	1	1
	**Panama**	**Panama**	**Panama**	1	1
	**Peru**	**Peru: Loreto**	**Iquitos**	89	16
			**Mazán**	10	10
			**Puerto America**	4	4
			**Santo Tomás**	10	9
		**Peru: Madre de Dios**	**Delta 1**	6	5
		**Peru: Piura**	**Sullana**	4	4
***AF***	**Ethiopia**	**Ethiopia: Amhara**	**Amhara**	19	17
			**Gondar**	28	11
		**Ethiopia: Oromia**	**Batu**	3	2
			**Bishoftu**	4	0
			**Jimma**	44	26
			**Oromia**	69	51
		**Ethiopia: SNNaP**	**South Nations Nationalities and** **Peoples' Region**	36	30
***WAS***	**Afghanistan**	**Afghanistan: Laghman**	**Laghman**	95	10
		**Afghanistan: Nangarhar**	**Jalalabad**	155	26
	**India**	**India**	**India**	2	2
			**India (returning traveller)**	1	0
		**India: Madhyapradesh**	**Indore (returning traveller)**	1	0
		**India: Maharashtra**	**Mumbai (returning traveller)**	2	1
		**India: Tamil Nadu**	**Chennai**	8	2
	**Iran**	**Iran**	**Iran**	15	5
	**Sri Lanka**	**Sri Lanka: Monaragala**	**Kataragama**	2	1
***WSEA***	**Thailand**	**Thailand: Kanchanaburi**	**Kanchanaburi**	20	20
		**Thailand: Tak**	**Mae Sot**	4	4
			**Tak**	42	40
			**Umphang**	11	5
			**Wangpha**	64	58
***ESEA***	**Cambodia**	**Cambodia: Battambang**	**Battambang**	9	9
		**Cambodia: Kampot**	**Kampot**	9	9
		**Cambodia: Koh Kong**	**Takavit**	2	1
		**Cambodia: Oddar Meanchey**	**Oddar Meanchey**	133	104
		**Cambodia: Pailin**	**Pailin**	1	1
		**Cambodia: Pursat**	**Pursat**	79	46
		**Cambodia: Ratanakiri**	**Ratanakiri**	3	2
	**Thailand**	**Thailand: Nakhon Ratchasima**	**Pakchong**	1	1
		**Thailand: Sisaket**	**Sisaket**	2	1
	**Vietnam**	**Vietnam**	**Vietnam**	1	0
		**Vietnam: Binh Phuoc**	**Binh Phuoc**	30	15
			**Dak O**	31	26
		**Vietnam: Gia Lai**	**Krong Pa**	34	28
		**Vietnam: Ho Chi Minh**	**Ho Chi Min**	42	33
			**Viet Anh Ward**	1	1
***MSEA***	**Malaysia**	**Malaysia: Sabah**	**Sabah**	108	73
		**Malaysia: Selangor**	**Klang**	1	0
	**Philippines**	**Philippines: Palawan**	**Rio Tuba**	6	3
***OCE***	**Indonesia**	**Indonesia: Papua**	**Papua Indonesia**	253	175
			**Papua Indonesia (returning** **traveller)**	29	16
	**Papua New** **Guinea**	**Papua New Guinea**	**Papua New Guinea**	8	1
			**Papua New Guinea (returning** **traveller)**	3	0
		**Papua New Guinea: East Sepik**	**East Sepik**	6	0
		**Papua New Guinea: Madang**	**Madang**	30	16
**Total**				1,777	1,031

**Table 3.** MalariaGEN studies contributing samples.

Study ID	Study title	Contact	Samples	Sites
**1044-PF-KH-FAIRHURST**	Genomics of parasite clearance and recrudescence rates in Cambodia	Thomas E Wellems twellems@niaid.nih.gov	82	Pursat (Cambodia), Ratanakiri (Cambodia)
**1046-PV-BR-FERRERIA**	Developing the *Plasmodium* *Vivax* Genome Variation Project with partners in Brazil	Marcelo Ferreira muferrei@usp.br	5	Brazil (Brazil)
**1047-PV-LK-** **KARUNAWEERA**	Developing the *Plasmodium* *Vivax* Genome Variation Project with partners in Sri Lanka	Nadira Karunaweera nadira@parasit.cmb.ac.lk	2	Kataragama (Sri Lanka)
**1049-PV-VN-BONI**	Developing the *Plasmodium* *Vivax* Genome Variation Project with partners in Vietnam	Tran Tinh Hien hientt@oucru.org	13	Binh Phuoc (Vietnam), Viet Anh Ward (Vietnam)
**1050-PV-PN-MUELLER**	Developing the *Plasmodium* *Viva*x Genome Variation Project with partners in Papua New Guinea	Ivo Mueller ivomueller@fastmail.fm	20	East Sepik (Papua New Guinea), Madang (Papua New Guinea)
**1052-PF-TRAC-WHITE**	Tracking Resistance to Artemisinin Collaboration (TRAC)	Elizabeth Ashley liz@tropmedres.ac	4	Bago (Myanmar), Binh Phuoc (Vietnam), Sisaket (Thailand)
**1098-PF-ET-GOLASSA**	The prevalence of asymptomatic carriage; emergence of parasite mutations conferring anti- malaria drug resistance; and G6PD deficiency in the human population, as possible impediments to malaria elimination in Ethiopia	Lemu Golassa lgolassa@gmail.com	88	Amhara (Ethiopia), Oromia (Ethiopia)
**1102-PF-MG-** **RANDRIANARIVELOJOSIA**	Genotyping *P. falciparum* and *P. vivax* in Madagascar	Milijaona Randrianarivelojosia milijaon@pasteur.mg	1	Maevatanana (Madagascar)
**1128-PV-MULTI-GSK**	A global survey of *P. vivax* genome variation in samples from two GSK phase 3 clinical trials of tafenoquine in Pv relapse/reinfection (trial names DETECTIVE and GATHER)	Anup Pingle anup. s.pingle@gsk.com	357	Bangkok (Thailand), Cali (Colombia), Gondar (Ethiopia), Ho Chi Min (Vietnam), Iquitos (Peru), Jimma (Ethiopia), Mae Sot (Thailand), Manaus (Brazil), Oddar Meanchey (Cambodia), Porto Velho (Brazil), Rio Tuba (Philippines), Umphang (Thailand)
**1154-PV-TH-PRICE**	Characterisation of drug resistance in *P. falciparum* and *P. vivax* populations from Indonesia and Thailand	Sarah Auburn Sarah.Auburn@menzies. edu.au	359	Papua Indonesia (Indonesia), Tak (Thailand), Wangpha (Thailand)
**1157-PV-MULTI-PRICE**	*P. vivax* SNP barcode for mapping parasite transmission and spread within and across borders: a vivaxGEN initiative	Sarah Auburn Sarah.Auburn@menzies. edu.au	667	Anhui (China), Antioquia (Colombia), Bangladesh (Bangladesh), Batu (Ethiopia), Bhutan (Bhutan), Binh Phuoc (Vietnam), Bishoftu (Ethiopia), Bolivar (Colombia), Choco (Colombia), Colombia (Colombia), Cordoba (Colombia), Córdoba (Colombia), Dak O (Vietnam), El Salvador (El Salvador), India (returning traveller) (India), Indore (returning traveller) (India), Iran (Iran), Jalalabad (Afghanistan), Kassala (Sudan), Klang (Malaysia), Krong Pa (Vietnam), Laghman (Afghanistan), Papua Indonesia (returning traveller) (Indonesia), Papua New Guinea (returning traveller) (Papua New Guinea), Pichimá (Colombia), Sabah (Malaysia), Santa Cecilia (Colombia), South Nations Nationalities and Peoples' Region (Ethiopia), Tierralta (Colombia)
**Total**			1,598	

**Table 4.** External studies contributing samples.

External Study ID	Manuscript title	Citation	Samples	Sites
**X0001-PV-MULTI-** **HUPALO2016**	Population genomics studies identify signatures of global dispersal and drug resistance in *Plasmodium vivax*	pubmed 27348298	195	Acrelândia (Brazil), Ampasimpotsy (Madagascar), Belem (Brazil), Brazil (Brazil), Buenaventura (Colombia), Carrillo (Mexico), Chennai (India), Choco (Colombia), Delta 1 (Peru), El Salvador (El Salvador), Frontera Hidalgo (Mexico), Huehuetán (Mexico), India (India), Iquitos (Peru), Kanchanaburi (Thailand), Laiza township (Myanmar), Madagascar (Madagascar), Madang (Papua New Guinea), Mauritania I (Mauritania), Mazán (Peru), Nicaragua (Nicaragua), North Korea (North Korea), Pailin (Cambodia), Pakchong (Thailand), Panama (Panama), Papua New Guinea (Papua New Guinea), Plácido de Castro (Brazil), Puerto America (Peru), Santo Tomás (Peru), Sullana (Peru), Takavit (Cambodia), Tapachula (Mexico), Tierralta (Colombia), Tumaco (Colombia), Tuxtla Chico (Mexico), Vietnam (Vietnam)
**X0002-PV-KH-** **PAROBEK2016**	Selective sweep suggests transcriptional regulation may underlie *Plasmodium* *vivax* resilience to malaria control measures in Cambodia	pubmed 27911780	78	Battambang (Cambodia), Kampot (Cambodia), Oddar Meanchey (Cambodia)
**X0009-PV-ET-LO**	Frequent expansion of *Plasmodium vivax* Duffy Binding Protein in Ethiopia and its epidemiological significance	pubmed 31509523	24	Jimma (Ethiopia)
**Total**			297	

All 1,598 samples contributed by MalariaGEN partners were sequenced at the Wellcome Sanger Institute using the Illumina platform. For the 297 samples published by other research groups, raw reads were obtained from the European Nucleotide Archive (PRJNA240356-PRJNA240533 and PRJNA295233). We mapped the sequence reads against the *P. vivax* P01 v1 reference genome and the median depth of coverage was 26x averaged across the whole genome and across all samples.

We constructed an analysis pipeline for variant discovery and genotyping, including stringent quality control filters as outlined in the Methods section. We discovered genome variation spanning 16% of the *P. vivax* genome (~4.5 million positions), with variation falling predominantly within non-coding regions ( Table 5). The majority of variation was in the form of SNPs (3,083,454), with the remaining 1,487,602 variants consisting of short indels, and occasionally more complex combinations of SNPs and indels that were at least three alleles. For the purpose of analysis, we excluded all variants in subtelomeric and internal hypervariable regions, mitochondrial and apicoplast genomes. A total of 945,649 SNPs (of which 911,901 were biallelic) and 358,335 indels (or SNP/indel combinations) passed this filtration step. The pass rates for SNPs and indels in coding regions (53%, 50%) were considerably higher than SNPs and indels in non-coding regions (22%, 18%). Short variant calls in both VCF and zarr format can be found via the data resource page ( https://www.malariagen.net/resource/30).

**Table 5.** Summary of discovered variant positions. We divide variant positions into those containing single nucleotide polymorphisms (SNPs) and non-SNPs (indels and combinations of SNPs and indels at the same position). We then further sub-divide each of these into those within exons (coding) and those in intronic or intergenic regions (non-coding). We further sub-divide SNPs into those containing only two alleles (bi-allelic) or those containing three or more alleles (multi-allelic). Discovered variant positions are unique positions in the reference genome where either SNP or indel variation was discovered by our analysis pipeline. Pass variant positions are the subset of discovered positions that passed our quality filters. Alleles per pass position shows the mean number of distinct alleles at each pass position; biallelic variants have two alleles by definition.

Type	Coding	Multi- allelic	Discovered variant positions	Pass variant positions	% pass	Alleles per pass position
SNP	Coding	Bi-allelic	827,373	440,222	53%	2.0
		Multi-allelic	40,311	17,111	42%	3.0
	Non-coding	Bi-allelic	1,927,558	471,679	24%	2.0
		Multi-allelic	288,212	16,637	6%	3.0
non-SNP	Coding		279,694	138,544	50%	3.4
	Non-coding		1,207,908	219,791	18%	3.4
Total			4,571,056	1,303,984	29%	2.4

As part of a detailed curation process, we removed samples with (i) unverified or incomplete sample collection information; (ii) evidence of co-infection with other *Plasmodium* species; (iii) more than one technical replicate or time course sampling (in which case we retained the sample for which the proportion of the genome covered was the greatest); (iv) low coverage, or (v) evidence of being an extreme genetic outlier. We directly compared data from MalariaGEN partner studies with those from other research groups in three locations where samples were available from both sources: Iquitos, Peru; Oddar Meanchey; Cambodia; Oromia, Ethiopia. We found no stratification by data source and no indications of significant biases. In total, we obtained 1,072 high-quality samples from 27 countries ( Table 1).

**Figure 1.**
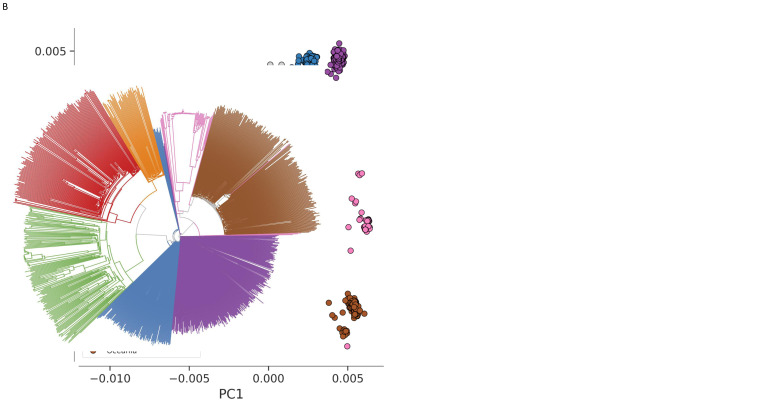
Population structure. ( **A**) First two components of a genome-wide principal coordinate analysis. Each point represents one of 1,072 QC pass samples coloured according to country groupings ( Table 1): Latin America (green, n=159); Africa (red, n=137); Western Asia (orange, n=47); West south-east Asia (blue; n=127); East south-east Asia (purple; n=277); Maritime south-east Asia (pink; n=76); Oceania (brown; n=208); Unassigned samples (grey; n=41). This shows the genetic separation of samples into seven distinct geographic clusters. This also shows that samples that have not been assigned to a region look distinct from those from the seven regions. After removal of the 41 unassigned samples we have an analysis set of 1,031 samples. ( **B**) Genome-wide unrooted neighbour-joining tree showing population structure across all sites from the seven regions (1,031 analysis set samples), with sample branches coloured as in **A**. This shows that maritime Southeast Asia has large numbers of very highly related parasites and clear relatedness between samples is also present in some samples from Latin America and Africa.

The genetic structure of the global parasite population largely reflects its geographic regional structure ^13^ as recapitulated by a principal component analysis of all samples based on their SNP genotypes ( Figure 1a). Here we divided samples into seven regional sub-populations of parasites with a high degree of geographic and genetic proximity (41/1,072 high-quality samples were not assigned to a regional sub-population giving a final analysis set of 1,031 samples). However, geography is not the only factor influencing the population structure, as different regions are impacted by a range of epidemiological and environmental effects, such as differences in transmission intensity, vector species and history of antimalarial drug usage. An example of this can be seen in the varying levels of regional population structure as illustrated with a neighbour-joining tree ( Figure 1b), with maritime Southeast Asia having large numbers of highly related parasites being the most striking example, as previously described ^17^. These regional classifications are intentionally broad, and therefore overlook many interesting aspects of local population structure. Sample information including partner study information, location and year of collection, ENA accession numbers, QC information and region assignment can be found on the resource page ( https://www.malariagen.net/resource/30).

Analysis of *F _WS_*, a measure of within-host diversity, shows that in all regions, the majority of samples have *F _WS_* > 0.95, which to a first approximation indicates that the infection is dominated by a clonal population of parasites. The proportions of such clonal samples were highest in Latin America (135/159, 85%), Maritime SE Asia (59/76, 78%) and Africa (102/137, 74%). In contrast, over 40% of samples from Eastern SE Asia (116/277, 42%) and Oceania (88/208, 42%) have *F _WS_* <0.95, indicating the presence of more complex infections. Interestingly, these results are in contrast to those in *P. falciparum* where complex infections are more common in Africa than in SE Asia ^18^, reflecting the different epidemiology of the two diseases. A file of *F _WS_* values for all QC pass samples can be found in the data resource ( https://www.malariagen.net/resource/30).

We genotyped tandem duplications using a novel two-stage process, where we first discovered base pair resolution breakpoints using a combination of read depth and split reads, and then genotyped samples at these discovered breakpoints using a combination of read depth and read pairs mapped in a tail-to-tail configuration. This hybrid approach allows us to assess the presence of known tandem duplications also in samples with low and uneven coverage or in complex infections. Compared to our previous release, the improved method now has the ability to distinguish unique breakpoints, as well as the distinct chromosomal fragment formations of these tandem duplication events.

We discovered seven pairs of distinct tandem duplication breakpoints in four different regions of the genome ( Table 6). Most breakpoints (5/7) were found to be homopolymer A/T repeats of >= 11 nucleotides in non-coding regions. The most common duplications were found around *dbp,* with two different sets of breakpoints previously described as the "Malagasy" and "Cambodian" duplications ^19, 20^. Interestingly, we found that the "Cambodian" duplication was common and widespread, with the highest proportion of samples in Africa, moderate frequencies in western/eastern Southeast Asia, and lower frequencies in maritime Southeast Asia/Oceania. In sharp contrast, the "Malagasy" duplication was only seen in African isolates.

We previously reported on a chromosome 14 duplication encompassing the gene PVP01_1468200 (conserved protein with unknown function previously annotated as PVX_101445) ^13^, and can now show that there are three different sets of breakpoints. The most common duplication is the short 3.5kb duplication which includes only the single gene PVP01_1468200. All three duplications are seen exclusively in Oceania. The tandem duplication calls for all samples can be found in the data resource ( https://www.malariagen.net/resource/30).

Molecular mechanisms of resistance in *P. vivax* are poorly understood ^21^, which restricts the ability to perform drug resistance sample classification to a very limited set of published and well-recognised genetic markers. We correspondingly classified all samples using a set of basic heuristics into four types of inferred drug resistance, with Table 7 summarising the frequency of samples classified as resistant in different geographical regions. Overall, we observed higher prevalence of inferred resistance in Southeast Asia and Oceania than elsewhere, with 18% samples in Western Southeast Asia inferred resistant to all three drugs considered (sulfadoxine, pyrimethamine and mefloquine). Notably, this is intended simply to provide analysis context, and cannot be considered as an accurate reflection of the current epidemiological situation.

The only combination therapy described here is sulfadoxine/pyrimethamine (SP), with SP resistant samples being classified into three overlapping types: (i) carrying the *dhfr* 117T allele, associated with pyrimethamine resistance; (ii) the *dhps* 383G allele, associated with sulfadoxine resistance; (iii) carrying the *dhfr* quadruple mutant, which is associated with SP failure. Amino acid calls at drug resistance loci, inferred drug resistance phenotypes and a document detailing heuristics used to infer these phenotypes can be found in the data resource ( https://www.malariagen.net/resource/30).

## Methods

### DNA sequencing

Standard laboratory protocols were used to determine DNA quantity and proportion of human DNA in each sample as previously described ^13^. 1,622 samples passing thresholds were put forward for whole genome Illumina paired-end sequencing. The majority of these were from MalariaGEN studies but 24 were sequenced at the Wellcome Sanger Institute in a collaboration between Julian Rayner and Eugenia Lo ^15^. A further 273 samples were downloaded from the SRA ^14, 16^. All 1,895 samples were analysed.

### Read mapping and coverage analysis

Reads mapping to the human reference genome were discarded before all analyses, and the remaining reads were mapped to the *P. vivax* P01 v1 reference genome ^22^ ( ftp://ftp.sanger.ac.uk/pub/project/pathogens/gff3/2016-10/PvivaxP01.genome.fasta.gz) using bwa mem ^23^ version 0.7.15 with –M parameter to mark shorter split hits as secondary.

Two of the steps in the pipeline (base quality score recalibration and variant quality score recalibration) require a set of known variants. For both of these steps we used the PASS variants from the PvGv 1.0 release. Given that the 1.0 release used the Sal1 reference, and the current release uses the P01 reference, we needed to convert the coordinates of the 1.0 release variants. We did this using the liftover tool, following the instructions at http://genomewiki.ucsc.edu/index.php/Minimal_Steps_For_LiftOver.

Various “bam improvement” steps were applied to the bwa outputs before further analyses. The Picard ( http://picard.sourceforge.net) tools CleanSam, FixMateInformation and MarkDuplicates were successively applied to the bam files of each sample, using Picard version 2.6.0. GATK version 3.8-0 base quality score recalibration was applied using only the core genome and the PASS variants from the PvGv 1.0 release as a set of known sites. All lanes from each library were merged to create library-level bam files, and then all libraries for each sample were merged to create sample-level bam files. The output of this stage was a set of 1,895 “improved” bam files, one for each sample.

Standard alignment metrics were generated for each sample using the stats utility from samtools version 1.2 ^24^. We also used GATK’s CallableLoci to determine the genomic positions callable in each sample ^25^. The following GATK parameters were used: --minDepth 5.

### Variant discovery and genotyping

We discovered potential SNPs and indels by running GATK’s HaplotypeCaller ^25^ version 3.8-0 independently across each of the 1,895 sample-level BAM files. The following GATK parameters were used: --emitRefConfidence GVCF --variant_index_type LINEAR --variant_index_parameter 128000 --max_alternate_alleles 6

This resulted in the creation of 1,895 GVCF files. We merged these for each of the 242 reference sequences (14 chromosomes, 1 apicoplast, 1 mitochondria and 226 short contigs) using GATK’s CombineGVCFs. Each of the 242 reference sequences was then genotyped using GATK’s GenotypeGCVFs with --max_alternate_alleles 6

The 226 separate VCF files for each short contig were concatenated into a single VCF using the concat command in bcftools v1.8.

### Variant filtering and annotation

SNPs and indels were filtered separately. For each class of variant, filtering was done in two stages: 1) Each variant was assigned a quality score using GATK’s Variant Quality Score Recalibration (VQSR) version 3.8-0. The tools VariantRecalibrator and ApplyRecalibration are used here, and 2) Regions of the genome which we previously identified as being enriched for errors ^13^ are masked out.

For SNPs, VariantRecalibrator was run using the PASS variants from the PvGv 1.0 release ^13^ as a training set with 15.0 as a prior, and the following parameters: -an QD -an FS -an SOR -an DP --maxGaussians 8 --MQCapForLogitJitterTransform 70. For indels we have no suitable training set so we used a “bootstrap” approach. We first identified a set of high quality indels from all indels discovered, by setting the same thresholds on the variables FS, MQ and QD as were used for SNPs in PvGv 1.0 (FS<=14.63418, MQ>=51.6, QD>=12.43). We then used this as a training set with a prior of 12.0 and the following parameters: -an QD -an DP -an SOR -an FS --maxGaussians 4 --MQCapForLogitJitterTransform 70. ApplyRecalibration was then run to assign each variant a quality score named VQSLOD. High values of VQSLOD indicate higher quality. Variants (both SNPs and indels) with a VQSLOD score ≤ 0 were filtered out.

Variants in the VCFs were annotated using a number of different methods. Functional annotations were applied using snpEff ^26 ^version 4.1, with gene annotations downloaded from GeneDB ^27^ at ftp://ftp.sanger.ac.uk/pub/project/pathogens/gff3/2018-05/PvivaxP01.noseq.gff3.gz. The following options were used with snpEff: -no-downstream -no-upstream -onlyProtein.

Genome regions were annotated using vcftools and masked if they were outside the core genome. The different genome regions can be found in file Pv4_regions.bed.gz available at the resource page. Variants in the apicoplast, mitochondrion and short contigs were annotated *Apicoplast*, *Mitochondrion* and *ShortContig* respectively and masked by adding these annotations to the FILTER column. Subtelomeric regions in the 14 chromosomal sequences were identified by determining the genes at the boundaries of the subtelomeric regions identified in the PvGv 1.0 release ^13^, and then using the coordinates of these same genes in the *P. vivax* P01 v1 reference sequence. Variants in these subtelomeric regions were annotated *SubtelomericHypervariable* and masked by adding this annotation to the FILTER column. Finally, the three internal chromosomal regions containing the *sera*, *msp3* and *msp7* families were annotated as *InternalHypervariable* and masked by adding this annotation to the FILTER column.

VCF files were converted to zarr format using scikit-allel v 1.2.0 ( https://github.com/cggh/scikit-allel) and subsequent analyses performed using the zarr files.

### Species identification

We identified species using nucleotide sequence from reads mapping to six different loci in the mitochondrial genome, using custom java code ( https://github.com/malariagen/GeneticReportCard). The loci were located within the *cox3* gene (PVP01_MIT02700), as described in a previously published species detection method ^28^. Alleles at various mitochondrial positions within the six loci were genotyped and used for classification ^18^. A sample is assigned a species if it matches at least two of the six loci. At any given locus, the sample is considered a match to a species only if all the positions at that locus carry the matching allele.

### Genetic distance

We calculate genetic distance between samples using biallelic coding SNPs that pass filters using a method previously described ^29^. For each SNP in sample *i* we calculate the non-reference allele frequency *f*_*i*_ as the proportion of reads that carry the non-reference allele. For clonal samples, *f*_*i*_ should be either 0 (for homozygous reference allele calls) or 1 (for homozygous alternative allele calls). For samples containing mixtures of different strains, we should expect fractional values of *f*_*i*_ for heterozygous calls. *f*_*i*_ is set to 0 if there are < 2 or <5% alternative allele reads, and likewise to 1 if there are < 2 or <5% reference allele reads. We do not calculate *f*_*i*_ when there were less than 5 reads in total. Genetic distance between sample 1 and 2 is calculated as *f*_1_ (1 – *f*_2_ ) + *f*_2_ (1 – *f*_1_). For each sample pair we calculate the mean genetic distance across all SNPs for which we have an estimate of *f*_*i*_ in each sample.

### Sample QC

We created a set of 1,072 QC pass samples after removing expected mislabelled, replicate, low coverage, mixed-species, and genetic outlier samples.

We first removed 107 samples where we had evidence that there might have been a mislabelling and hence are not sure of the true identity of the samples.

We calculated genome callability of each sample using GATK CallableLoci with a minimum depth of 5. Where we had multiple samples from the same individual, we removed samples with lower callability to leave a single sample for each individual in the QC pass set. This removed 145 samples. A further 548 samples with callability <50% were also removed.

We removed a further 22 samples from the analysis set that were identified as containing mixed species. We note that many of these samples appeared as outliers on neighbour-joining trees before their removal (data not shown).

Finally, we noted that sample PNG_chesson had much higher median genetic distance to other samples than all other samples. The median genetic distance from PNG_chesson to other samples was 0.055, whereas the median genetic distances to other samples for all other samples was between 0.18 and 0.24. We removed PNG_chesson from the final analysis set as a genetic outlier. The final analysis set contained 1,072 QC pass samples.

### Population structure and characterisation

The matrix of genetic distances was used to generate neighbour-joining trees and principal coordinates. Neighbour-joining trees (NJTs) were produced using the nj implementation in the R package ape. Principal coordinate analysis (PCoA) was performed using scikit-bio v0.5.5. Based on these observations we grouped the samples into seven geographic regions: Latin America, Africa, West Asia, the western part of Southeast Asia, the eastern part of Southeast Asia, maritime Southeast Asia and Oceania, with samples assigned to region based on the geographic location of the sampling site. 17 samples from returning travellers were assigned to region based on the reported country of travel. 41 QC pass samples from countries with small numbers of samples that did not cluster with those from one of these seven regions were left unassigned, so the population genetic analyses in this paper are based on 1,031 analysis set samples from the seven regions. *F _WS_* was calculated using custom python scripts using the method previously described ^30^.

### Tandem duplication genotyping

We genotyped tandem duplications using a novel two-stage process where we first discovered base pair resolution breakpoints using a combination of read depth and split reads and then genotyped samples at these discovered breakpoints using a combination of read depth and read pairs mapped in a tail-to-tail configuration. The outline algorithm for discovering breakpoints is as follows. For each QC pass sample sequenced from genomic DNA (not from material that underwent whole genome amplification or hybrid selection) that passes QC:

1. Calculate normalised coverage for every 300bp non-overlapping window as coverage of window/median coverage of all core genome windows
2. Determine putative increases in copy number by running an HMM across normalised coverage bins
3. Discard discovered regions shorter than 3kb
4. Automatically determine breakpoints for each putative tandem duplication using a custom python script that searches for clipped reads in read pairs where each read in the pair maps within 1kb of the breakpoints identified by the coverage HMM
5. For each pair of breakpoints, determine the maximal common sequence around the sequence, e.g. expand any homopolymer sequences to the ends of the homopolymer repeats
6. Discard any putative tandem duplication where breakpoints could not be determined

We then identify the unique set of breakpoint regions across all samples, and for breakpoint regions that overlap, determine the maximal region that is included in all. This set of breakpoint regions ( Table 6) is then used in the genotyping stage. Here, for each sample, the outline algorithm is as follows

**Table 6.** Geographic patterns of tandem duplications. Breakpoint IDs are shown in the first column (Duplication name) and can be used to match to the per sample breakpoints in the data release. Breakpoints are generally poly-A or poly-T repeats and First and Second breakpoints columns show the start positions and sequence of the breakpoint sequences in the reference genome (A _18_ denotes a poly-A sequence of 18 bases, i.e. AAAAAAAAAAAAAAAAAA). Length column shows the length in bp between the inner ends of the breakpoints. Percentages in Frequency (red) show the proportion of samples which could be genotyped that have a duplication (copy number >= 1.5). LAM=Latin America, AF=Africa, WAS=West Asia, WSEA=West south-east Asia, ESEA=East south-east Asia, MSEA=Maritime south-east Asia, OCE=Oceania, *n*=range of numbers of samples that could be genotyped at the different duplications.

					Frequency						
Duplication name	Chrom	Length	First breakpoint	Second breakpoint	LAM *n*=25–28	AF *n*=112–114	WAS *n*=11–14	WSEA *n*<=91–105	ESEA *n*=198–220	MSEA *n*=59–63	OCE *n*=116–133
**DBP_Cambodian**	PvP01_06_v1	7,333	980,472 A _18_	987,823 A _15_	0%	73%	0%	29%	35%	7%	5%
**DBP_Malagasy**	PvP01_06_v1	8,179	980,472 A _18_	988,669 A _22_	0%	10%	0%	0%	0%	0%	0%
**PvP01_09**	PvP01_09_v1	44,831	392,555 GG	437,388 GG	0%	0%	0%	0%	<1%	0%	0%
**MDR1**	PvP01_10_v1	38,134	468,190 A _15_	506,339 A _18_	0%	0%	0%	19%	0%	0%	0%
**PVP01_1468200_long**	PvP01_14_v1	26,452	2,894,706 GAAG	2,921,162 GAAG	0%	0%	0%	0%	0%	0%	3%
**PVP01_1468200_medium**	PvP01_14_v1	11,798	2,901,140 A _11_	2,912,949 A _30_	0%	0%	0%	0%	0%	0%	1%
**PVP01_1468200_short**	PvP01_14_v1	3,517	2,903,559 T _17_	2,907,093 T _16_	0%	0%	0%	0%	0%	0%	26%

**Table 7.** Frequency of different sets of polymorphisms putatively associated with drug resistance in samples from different geographical regions. All samples were classified into different types of drug resistance based on published genetic markers, and represent best attempt based on the available data. Each type of inferred resistance was considered to be either present, absent or unknown for a given sample. For each inferred resistance type, the table reports: the genetic markers considered; the drug they are associated with; the proportion of samples in each region classified as inferred resistant out of the samples where the type was not unknown. The number of samples classified as either resistant or not resistant varies for each type of inferred resistance considered (e.g. due to different levels of genomic accessibility); numbers in brackets in the header report the minimum and maximum number analysed while the exact numbers are reported in brackets below each percentage. SP: sulfadoxine-pyrimethamine; treatment: SP used for the clinical treatment of uncomplicated malaria. Details of the rules used to infer resistance status from genetic markers can be found on the resource page at www.malariagen.net/resource/30.

Marker	Associated with resistance to	Latin America (n=26–158)	Africa (n=114–137)	West Asia (n=14–46)	Western Southeast Asia (n=101–127)	Eastern Southeast Asia (n=220–276)	Maritime Southeast Asia (n=63–76)	Oceania (n=132–205)
***dhfr*** **117T**	Pyrimethamine	1% (1/158)	0% (0/137)	0% (0/46)	89% (110/124)	0% (0/276)	93% (69/74)	77% (138/180)
***dhps*** **383G**	Sulfadoxine	55% (84/152)	23% (31/134)	13% (6/46)	100% (127/127)	89% (230/259)	95% (72/76)	88% (181/205)
***mdr1*** **2+ copies**	Mefloquine	0% (0/26)	0% (0/114)	0% (0/14)	18% (18/101)	0% (0/220)	0% (0/63)	0% (0/132)
***dhfr*** **quadruple** **mutant**	SP (treatment)	0% (0/158)	0% (0/137)	0% (0/45)	88% (103/117)	0% (0/276)	93% (64/69)	77% (131/171)

7. Calculate normalised coverage for every 300bp non-overlapping window as coverage of window/median coverage of all core genome windows
8. For each breakpoint region, set initial copy number to median of normalised coverage across 300bp windows in that region, rounded to the nearest integer
9. For each set of breakpoints, we determine the number of reads that are in 600bp window starting half a read length before the first breakpoint, and the proportion of these for which both a) the mate is within a 600bp window before the second breakpoint and b) the pair are in face-away orientation
10. If the number of reads in 9. is greater than 100 or the proportion is greater than zero but less than 2.5%, we assume the call is undetermined and set the copy number for the region to missing
11. If the number of reads in 9. is greater than 100, and the proportion of face-away is greater than 2.5%, but the initial copy number determined in 8, is 1, we assume there is a heterozygous duplication, and set the copy number to 1.5

We carried out the following analyses that show that the above algorithm is likely to be a reliable method for calling tandem duplications in *P. vivax* whole genome data. Firstly, we note that in all cases, where we found a copy number >= 2 using read depth, we also found read pairs consistent with at least one of the sets of breakpoints, i.e. we have exact breakpoints for all tandem duplication genotypes. Secondly, for cases where we found a copy of number one using read depth, but found evidence of breakpoint read pairs, the copy number was generally between one and two, and all but one sample had an *F _WS_* value < 0.95 indicating mixed infections, and as such heterozygote calls (copy number 1.5) appear to be appropriate. Finally, a subset of samples have previously been assessed for *dbp* using qRT-PCR, and our calls were highly concordant with those results (data not shown) ^15, 20^.

### SNP genotypes at drug resistance mutations and samples classification

We extracted genotypes at loci implicated in drug resistance from the VCF files (GT fields). At some loci we could not use amino acid changes annotated in the VCF files because a) the codon contains multiple variable positions, b) some positions within the codon have multi-allelic variants, or, c) as is the case for *dhfr* and *dhps*, there are combinations of multiple SNPs and indels. We developed a custom python script to call amino acids at selected loci by first determining the reference amino acids and then, for each sample, applying all variations using the GT field of the VCF file. Where a locus included multiple heterozygous variants, we used the PID and PGT VCF fields to phase the variants where possible. We calculated allele frequencies assuming a frequency of 1.0 for homozygous alternative calls, and 0.5 for heterozygous calls.

The amino acid and copy number calls generated were used to classify all samples into different types of drug resistance. Our methods of classification were heuristic and based on the available data and current knowledge of the molecular mechanisms. Each type of resistance was considered to be either present, absent or unknown for a given sample. The procedure used to map genetic markers to inferred resistance status classification is described in detail for each drug in the accompanying data release ( https://www.malariagen.net/resource/30).

## Data availability

### Underlying data

This project contains the following underlying data that are available as an online resource: https://www.malariagen.net/resource/30. Data are also available from Figshare.

Figshare: Supplementary data to: An open dataset of *Plasmodium vivax* genome variation in 1,895 worldwide samples. https://doi.org/10.6084/m9.figshare.19367876.

- Study information: Details of the 11 contributing partner studies, and 3 external studies, including description, contact information and key people.
- Sample provenance and sequencing metadata: sample information including partner study information, location and year of collection, ENA accession numbers, and QC information for 1,895 samples from 27 countries.
- Measure of complexity of infections: characterisation of within-host diversity (F _WS_) for 1,072 QC pass samples.
- Drug resistance marker genotypes: genotypes at known markers of drug resistance for 1,895 samples, containing amino acid and copy number genotypes at 3 loci: dhfr, dhps, mdr1.
- Inferred resistance status classification: classification of 1,072 QC pass samples into different types of resistance to 4 drugs or combinations of drugs: pyrimethamine, sulfadoxine, mefloquine, and sulfadoxine-pyrimethamine combination.
- Drug resistance markers to inferred resistance status: details of the heuristics utilised to map genetic markers to resistance status classification.
- Tandem duplication genotypes: genotypes for tandem duplications discovered in four regions of the genome.
- Genome regions and Genome regions index: a bed file classifying genomic regions as core genome or different classes of non-core genome in addition to tabix index file for genome regions file.
- Short variants genotypes: Genotype calls on 4,571,056 SNPs and short indels in 1,895 samples from 27 countries, available both as VCF and zarr files.

Data are available under the terms of the Creative Commons Attribution 4.0 International license (CC-BY 4.0).

## Consent

All samples in this study were derived from blood samples obtained from patients with *P. vivax* malaria, collected with informed consent from the patient or a parent or guardian. At each location, sample collection was approved by the appropriate local and institutional ethics committees. The following local and institutional committees gave ethical approval for the partner studies: Human Research Ethics Committee, Walter and Eliza Hall Institute, Australia; Human Research Ethics Committee of NT Department of Health and Families and Menzies School of Health Research, Darwin, Australia; Islamic Republic of Afghanistan Ministry of Public Health Institutional Review Board, Afghanistan; ICDDR,B Ethical Review Committee, Bhutan; Research Ethics Board of Health, at the Ministry of Health in Bhutan; Institutional Review Board of the Institute of Biomedical Sciences, University of São Paulo, Brazil; National Ethics Committee for Health Research, Phnom Penh, Cambodia; Institutional Review Board of Jiangsu Institute of Parasitic Diseases, Wuxi, China; Comite Instiucional de Etica de Investigaciones en Humanos, Colombia; Comite de Bioetica Instituo de Investigaciones Medicas Facultad de Medicina Universidad de Antioquia, Colombia; Armauer Hansen Research Institute Institutional Review Board, Ethiopia; Addis Ababa University College of Natural Sciences, Ethiopia; Addis Ababa University, Aklilu Lemma Institute of Pathobiology Institutional Review Board, Ethiopia; National Research Ethics Review Committee of Ethiopia; Eijkman Institute Research Ethics Committee, Jakarta, Indonesia; Comité National d'Ethique auprès du Ministère de la Santé Publique, Madagascar; National Ethics Committee for Health Research, Lao Peoples' Democratic Republic; Research Review Committee of the Institute for Medical Research and the Medical Research Ethics Committee (MREC), Ministry of Health, Malaysia; The Government of the Republic of the Union of Myanmar, Ministry of Health, Department of Medical Research, Lower Myanmar, Myanmar; Papua New Guinea Institute of Medical Research Institutional Review Board, the Medical Research Advisory Committee of Papua New Guinea; Ethics Review Committee, Faculty of Medicine, University of Colombo, Sri Lanka; Ethics Committee, Faculty of Tropical Medicine, Mahidol University, Bangkok, Thailand; Oxford Tropical Research Ethics Committee, Oxford, UK; Institutional Review Board, National Institute of Allergy and Infectious Diseases, Bethesda, Maryland, USA; Scientific and Ethical Committee of the Hospital for Tropical Diseases in Ho Chi Minh City, Vietnam; The Ministry of Health Evaluation Committee on Ethics in Biomedical Research, Vietnam;
