# Supplementary material for: An open dataset of Plasmodium vivax genome variation in 1,895 worldwide samples
Source: Wellcome Open Res. 2022 Apr 14;7:136. [Version 1] doi: 10.12688/wellcomeopenres.17795.1 (PMC9127374; doi:10.12688/wellcomeopenres.17795.1)
Supplement: Peer review history

# Peer review: Reviewer response for version 1

**DOI:** 10.21956/wellcomeopenres.19696.r50006

**Published:** 2022-05-23

**Authors:** Jonathan J. Juliano

**Affiliations:** 1 Division of Infectious Diseases, University of North Carolina School of Medicine, Chapel Hill, NC, USA

The manuscript “An open dataset of *Plasmodium vivax* genome variation in 1,895 worldwide samples” represents a brief analysis of the genetic variation of currently available *P*. *vivax* genomic data. The samples are drawn largely from new MalariaGen and VivaxGen projects but include 3 other large population studies previously published. The investigators carefully re-analyzed all of the data to provide uniformity and generate a basic resource of parasite genetic diversity for the malaria community. These data and analyses are reportedly available in the public space on the MalariaGen resources pages. Overall, the analysis pipelines are appropriate and should yield accurate information concerning the underlying genome variation. The manuscript reports basic analysis (variant calls, basic population structure, basic allele frequencies of putative drug resistance polymorphisms). As they note, this leaves a lot of potentially interesting analyses to be done yet. Overall, this is a well done project and is well written. The summary of the data and methods of data generation are generally very clear. This is clearly a useful resource for the malaria community moving forward. I have a few minor comments that should be addressed by the authors:

  

1. I know this might seem nit picky, but the fact is that they really are presenting data on 1,072 samples, so the title is misleading. I would consider changing the title and abstract to reflect the analyzed samples.
2. The tandem duplication genotyping method is intriguing. Based on the process I would assume the result is very conservative in calling duplications. It would be nice to allow for this to be compared to other methods used. The code for this analysis does not seem to be publicly available (or at least is not cited as to where it is in the manuscript) which it really should be to allow other investigators to evaluate it relative to their work.

Are sufficient details of methods and materials provided to allow replication by others?

Yes

Is the rationale for creating the dataset(s) clearly described?

Yes

Are the datasets clearly presented in a useable and accessible format?

Yes

Are the protocols appropriate and is the work technically sound?

Yes

Reviewer Expertise:

Malaria genomics, epidemiology, molecular biology

I confirm that I have read this submission and believe that I have an appropriate level of expertise to confirm that it is of an acceptable scientific standard.

# Peer review: Reviewer response for version 1

**DOI:** 10.21956/wellcomeopenres.19696.r50005

**Published:** 2022-05-17

**Authors:** Daniel E. Neafsey

**Affiliations:** 1 Department of Immunology and Infectious Diseases, Harvard T.H. Chan School of Public Health, Boston, MA, USA; 2 Infectious Disease and Microbiome Program, Broad Institute of MIT and Harvard, Cambridge, Cambridge, MA, USA

This Data Note describes a large aggregation of existing and newly generated whole genome sequence data for the *Plasmodium vivax* malaria parasite, spanning many countries around the world. This resource will clearly have significant utility to the *P. vivax *research community for the foreseeable future. The report is clearly written and the methods are well described. I have several small suggestions to clarify or improve the manuscript, recognizing that follow-up manuscripts are likely to explore various biological themes in greater detail.

1. Table 1 depicts extreme variation in the % of samples that pass QC for analysis from different countries. Can the factors associated with high vs. low QC rates be described from this large aggregation of data, in order to help other investigators collect/store/extract DNA amenable to whole genome sequencing? It is notable that of the 1,895 samples mentioned in the title, only approximately half (1072) yielded data of sufficient quality for analysis.
2. The regions depicted in various colors in the NJ tree and PCA of Figure 1 made me wish for a small map with countries color-coded to accompany those plots. While Table 2 lists countries associated with each geographic region, it is tricky to remember which countries are encompassed by 'Western Asia' vs. 'Western Southeast Asia' without consulting Table 2 repeatedly.
3. The Figure 1 legend and article text use the term 'related' or 'relatedness' to describe parasite populations exhibiting shallow branch lengths in the NJ tree. However, given the tree was built using a metric of identity by state (IBS) at variant positions, rather than an actual estimate of relatedness (eg IBD), then it would be more appropriate to refer to such parasite populations as 'highly similar' instead of 'highly related.' I agree that the highly similar parasite populations are likely also highly related, but it is useful to use these terms precisely to avoid confusion in other contexts where the distinction among them is more vital.
4. On page 16 PNG_chesson is described as having a much higher genetic distance to other parasite samples, yet the value cited (0.055) is lower (rather than higher) than the typical genetic distance between all other parasite samples (0.18-0.24). This is confusing and requires clarification.

Are sufficient details of methods and materials provided to allow replication by others?

Yes

Is the rationale for creating the dataset(s) clearly described?

Yes

Are the datasets clearly presented in a useable and accessible format?

Yes

Are the protocols appropriate and is the work technically sound?

Yes

Reviewer Expertise:

Malaria genomics, evolution

I confirm that I have read this submission and believe that I have an appropriate level of expertise to confirm that it is of an acceptable scientific standard.
